# Pathophysiological Mechanisms in Neurodevelopmental Disorders Caused by Rac GTPases Dysregulation: What’s behind Neuro-RACopathies

**DOI:** 10.3390/cells10123395

**Published:** 2021-12-02

**Authors:** Marcello Scala, Masashi Nishikawa, Koh-ichi Nagata, Pasquale Striano

**Affiliations:** 1 Department of Neurosciences, Rehabilitation, Ophthalmology, Genetics, Maternal and Child Health, University of Genoa, 16132 Genoa, Italy; strianop@gmail.com; 2 Pediatric Neurology and Muscular Diseases Unit, IRCCS Istituto Giannina Gaslini, 16147 Genoa, Italy; 3 Department of Molecular Neurobiology, Institute for Developmental Research, Aichi Developmental Disability Center, 713-8 Kamiya, Kasugai 480-0392, Japan; mnishikawa@inst-hsc.jp (M.N.); knagata@inst-hsc.jp (K.-i.N.); 4 Department of Neurochemistry, Nagoya University Graduate School of Medicine, 65 Tsurumai-cho, Nagoya 466-8550, Japan

**Keywords:** Rho family guanosine triphosphatases, RAC1, RAC3, cytoskeletal dynamics, neuronal development, neurodevelopmental disorder, neuro-RACopathies, TRIO, DOCK, PAK

## Abstract

Rho family guanosine triphosphatases (GTPases) regulate cellular signaling and cytoskeletal dynamics, playing a pivotal role in cell adhesion, migration, and cell cycle progression. The Rac subfamily of Rho GTPases consists of three highly homologous proteins, Rac 1–3. The proper function of Rac1 and Rac3, and their correct interaction with guanine nucleotide-exchange factors (GEFs) and GTPase-activating proteins (GAPs) are crucial for neural development. Pathogenic variants affecting these delicate biological processes are implicated in different medical conditions in humans, primarily neurodevelopmental disorders (NDDs). In addition to a direct deleterious effect produced by genetic variants in the RAC genes, a dysregulated GTPase activity resulting from an abnormal function of GEFs and GAPs has been involved in the pathogenesis of distinctive emerging conditions. In this study, we reviewed the current pertinent literature on Rac-related disorders with a primary neurological involvement, providing an overview of the current knowledge on the pathophysiological mechanisms involved in the neuro-RACopathies.

## 1. Introduction

Twenty small Rho family guanosine triphosphatases (GTPases) have been identified in humans, classified in eight major subfamilies based on structural and biological properties: Rho-, Rac-, Cdc42-, RhoU/RhoV-, Rnd-, RhoD/R-, hoF-, RhoBTB-, and RhoH subfamilies [1,2]. As relevant key regulators of cytoskeletal dynamics and intracellular signaling, Rho family guanosine triphosphatases (GTPases) are crucial for the regulation of several fundamental biological processes in various cell types, including cell cycle progression, gene transcription, and cell morphology, motility, and polarity [3]. Furthermore, the direct modulation of Rho GTPase activity has emerged as a relevant contributor to the maintenance of genomic stability, which is implicated in a variety of human conditions [4].

**Figure 1:**
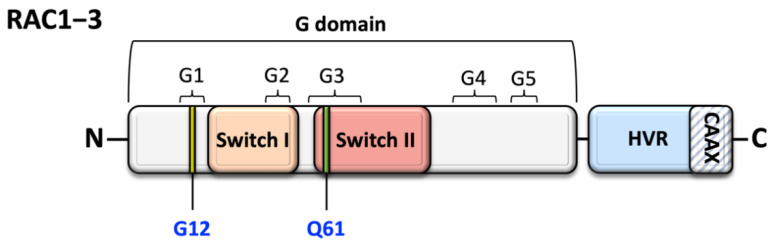
Schematic structure of members of Rac subfamily of Rho GTPases. The main functional domain is the G domain, which mediates GDP/GTP binding and GTP hydrolysis. In addition to the highly conserved residues Glycine 12 (G12) and Glutamine 61 (Q61), this region includes Switch I and II domains, which contain the consensus binding sites for the GAPs, GEFs, regulatory proteins, and effectors. Switch I and II are directly involved in the formation of the active GTP-bound state and undergo a structural rearrangement upon nucleotide exchange and hydrolysis, which is an essential step to initiate intracellular signaling cascades. The C-terminal hypervariable region (HVR) contains the terminal CAAX box, a consensus sequence where posttranslational modifications occur to determine the subcellular localization of the protein.

The RAC subfamily is part of the typical group of Rho GTPases, together with Rho- and CDC42-subfamilies, meaning that the members of this subfamily undergo classical GTPase cycle [5]. In particular, they act as a molecular switch, cycling between GTP-bound (active) and guanosine diphosphate (GDP)-bound (inactive) states, in a cycle which is strictly regulated by three groups of regulatory proteins with a very specific functional role: GTPase-activating proteins (GAPs), guanine nucleotide-dissociation inhibitors (GDIs), and guanine nucleotide exchange factors (GEFs) [6]. GDP/GTP binding and GTP hydrolysis are mediated by the highly conserved G domain (Figure 1).

This domain consists of five motifs with a conserved sequence (G1–5) that are involved in nucleotide binding and hydrolysis [7,8]. Two of these regions in particular, named Switch I (G2) and Switch II (G3), undergo a complex conformational rearrangement acting as an ‘’off-on’’ signal [7,9]. The conformational change of two crucial functional regions known as Switch I and II, conserved among different members of the RAC subfamily, is directly involved in the formation of the active GTP-bound state, offering a platform for selective interaction with a variety of downstream effectors, which in turn can initiate intracellular signaling cascades [10,11]. As discussed further below, these domains are mutational hotspot in Rac1- and Rac3-related disorders, in which causative pathogenic variants affect the function of Switch I or II in an activating or context-dependent way manner. The membrane anchorage and, thus, the subcellular localization of Rac proteins relies instead on a series of posttranslational modifications occurring at a cysteine residue in the CAAX motif (where C stands for cysteine, A for any aliphatic amino acid, and X for any amino acid), such as isoprenylation (geranylgeranylation or farnesylation), endoproteolysis, and carboxyl methylation [12].

**Figure 2:**
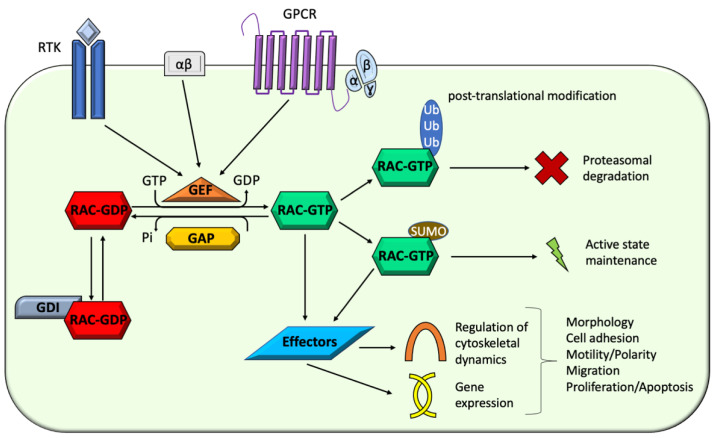
Schematic drawing illustrating the GTPase cycle, its regulatory mechanisms, and functional consequences. RAC GTPases cycle between an inactive GDP-bound form (Rac-GDP) and an active GTP-bound form (Rac-GTP). Transmembrane receptors (e.g., G-protein coupled receptors (GPCRs) or Receptor Tyrosine Kinases (RTKs)) mediate external signals converging on the GEFs, which promote Rac activation. The activated form (Rac-GTP) interacts with downstream effectors implicated in the regulation of cytoskeletal dynamics and gene expression regulation. Ancillary mechanisms modulating RAC signaling level involves active form stabilization by Sumoylation or ubiquitin-mediated proteasomal degradation and GDIs-mediated inactive form sequestration. Through different downstream effectors, Rac signaling converge into the regulation of cytoskeletal dynamics and gene expression.

While GEFs promote the dissociation of GDP and the binding of GTP, resulting in the activated state of the protein, GAPs stimulate the intrinsic GTPase activity and the GTP hydrolysis, thus terminating the signaling and completing the cycle [13,14]. Acting as negative regulators, GDIs are instead essential to regulate the amount of available GTPases and also modulate their targeting to intracellular compartments [1,2]. Once in the active state, the GTPases can activate several distinct downstream effectors, ranging from actin-related proteins to kinases (Figure 2) [15].

Three members are included in the RAC subfamily of Rho GTPases, RAC1–3, sharing around 90% of their sequences and mostly differing between each other in the carboxy-terminal ends [5]. The distribution of these proteins is quite restricted for RAC2, specifically expressed in hematopoietic cell lines, and RAC3, abundant in the developing and adult nervous system, whereas RAC1 is ubiquitously expressed [16,17,18,19]. Pathogenic variants affecting the function of Rac proteins have been identified as causative of different disorders, including immunodeficiencies and neurodevelopmental conditions (Table 1).

**Figure 3:**
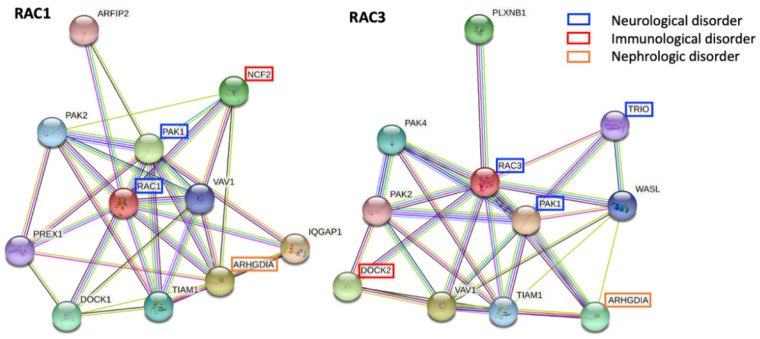
Predicted interactions of RAC1 and RAC3 with other proteins according to STRING (https://string-db.org, accessed on 19 October 2021). Some of these interactors are already associated with monogenic human conditions with or without a primary neurological involvement. Among RAC1 interactors, PAK1 (OMIM * 602590) is associated with a NDD with microcephaly, NCF2 (OMIM * 608515) with a chronic granulomatous disorder, and ARHGDIA (OMIM * 601925) with nephrotic syndrome. While PAK1 and ARHGDIA are common interactors to both RAC1 and RAC3, more specific RAC3 interactors with a known role in the pathogenesis of human conditions include TRIO (OMIM * 601893), linked to an autosomal dominant form of intellectual disability with micro- or macro-cephaly, and DOCK2 (OMIM * 603122), whose biallelic variants cause an immunodeficiency disorder.

In addition to specific regulators and effectors, Rac proteins are predicted to interact with several proteins with a relevant biological role. Some of these interactors are already associated with a human condition, with or without a primary neurological involvement, according to the Online Mendelian Inheritance in Man (OMIM) database (https://www.omim.org, accessed on 19 October 2021) (Figure 3).

Among RAC1 and RAC3 interactors, some have been already associated with human phenotypes, both neurological and extra-neurological. In the first group, there are PAK1 (OMIM * 602590) and TRIO (OMIM * 601893). PAK1 is a common interactor to RAC1 and RAC3, and is associated with an autosomal dominant NDD known as intellectual developmental disorder with macrocephaly, seizures, and speech delay (IDDMSSD, OMIM # 618158). TRIO (OMIM * 601893) is instead a more specific interactor of RAC3 and it is linked to autosomal dominant intellectual disability 44 with microcephaly (OMIM # 617061) or macrocephaly (OMIM # 618825). Extra-neurological phenotypes include immunological and renal diseases. In particular, autosomal recessive chronic granulomatous disease 2 (OMIM # 233710) is associated with the RAC1 interactor NCF2 (OMIM * 608515), and the immunodeficiency 40 (OMIM # 616433) is linked to the RAC3 interactor DOCK2 (OMIM * 603122). ARHGDIA (OMIM * 601925) interacts instead with both RAC1 and RAC3, and is linked to autosomal recessive nephrotic syndrome 8 (OMIM # 615244). The possible role of other interacting proteins in human disorders remains to be elucidated.

In this review, we provide an overview of the current knowledge on the pathophysiological mechanisms involved in RAC-related disorders with neurodevelopmental manifestations (neuro-RACopathies), especially focusing on the relevance of the functional disruption of these Rho GTPases and their regulatory proteins in neuronal cells. The understanding of the pathophysiological links underlying these conditions is of particular interest considering the growing interest on the biological significance of abnormal Rac signaling in human disease and the report of additional cohorts of affected individuals.

## 2. RAC1

### 2.1. RAC1 Structure and Function

Several studies have provided scientific evidence in favor of a critical role for RHO family GTPases in neuronal development, also entailing the pathogenic implications of a disrupted GTPase signaling in the pathophysiology of neurodevelopmental disorders (NDDs) [3,20,21,22,23]. Rac1 and Rac2 were the first members of the Rac subfamily to be characterized, with Rac1 being identified as a 21,450 dalton protein substrate for ADP-ribosylation by the C3 component of botulinum toxin [24]. Rac1 is ubiquitously expressed from the early embryonic stage [16]. A high sequence similarity (88–92% identity) is typical of the Rac subfamily of RHO GTPases, which mostly diverge in the last 10 carboxy-terminal residues [21]. Two Rac1 transcripts (1.2 and 2.5 kb) are ubiquitously expressed in a tissue-specific manner, based on two competing alternative polyadenylation sites [25]. Alternative splicing events involving the additional exon 3b, lacking in RAC2, lead to the generation of the constitutively active mutant Rac1b, which plays a crucial role in lamellipodia formation in fibroblasts [25].

The pivotal role of Rac1 in several fundamental biological processes is well exemplified by the involvement of this RHO GTPase as a crucial modulator of the cytoskeleton, with relevant implications for phagocytosis, adhesion, and migration [25,26]. Rac1 plays specific roles in the regulation of growth, maintenance, and retraction of growth cones (GCs), which are highly motile structures used by differentiating neurons to explore the surrounding environment and develop proper contacts during brain development [27]. GCs consist of the combination of a flat extension located at the tip of neurites, the lamellipodium, from which finger-like structures named filopodia emerge [28]. Molecular motors (e.g., myosins and dyneins) and several specialized proteins cooperate to regulate actin filaments polymerization, representing the main drive of GC protrusion [19,28]. Actin related protein 2/3 complex (Arp2/3) regulates actin filaments branching and coordinate filopodia formation and dynamics [29,30,31]. This essential protein is activated by Wiskott-Aldrich syndrome protein (WASP) family members, that are downstream effectors of Rac pathways [32,33]. In particular, Arp2/3-mediated actin polymerization is favored by the release of active WAVE (WASP family Verprolin Homology Domain-containing protein) from the homologous complex [32,33]. Therefore, Rac1 plays a crucial role in the regulation of growth speed, directionality, and persistence of lamellipodia through the modulation of Arp2/3-driven actin branching and polymerization [28]. Indeed, Rac1-deficient fibroblast cell lines lacking detectable levels of Rac2 and Rac3 were found to lack lamellipodia and this phenotype was rescued by either member of the Rac subfamily [34]. Despite the Rac1 deficiency not impairing the formation of focal adhesions, it significantly affected their assembly [34]. Accordingly, Rac1 signaling is essential for cell migration and lamellipodia protrusion, but not for filopodia-mediated cell spreading [34].

The regulation of neuronal proliferation by progenitor cells significantly relies on the Rac1 function. During corticogenesis, neurons are generated from a pool of progenitors lining the lateral ventricles (ventricular zone, VZ) whereas glial cells derive from a region positioned just above the VZ, named the subventricular zone (SVZ) [35,36]. Schematically, neuroblasts migrate toward the pial surface through a four-step radial migration process involving the maintenance and departure of progenitor cells from VZ, multipolar migration and transition to bipolar cells, radial glia-regulated locomotion, terminal translocation, and dendritic maturation [20,37,38]. Rac1 is required for the proper proliferation and differentiation of SVZ progenitors, and it plays an essential role in supporting the survival of both VZ and SVZ progenitors [36,37,38,39]. Indeed, the loss of Rac1 in the forebrain leads to a specific decrease in the proliferation and an increase in the cell cycle exit of SVZ progenitors, associated with their premature differentiation [37,39]. Rac1 mutants lack a clear delineation between SVZ and VZ, which appear as a unique compact region of prematurely differentiating mixed cells with decreased Cyclin D2 expression [37].

Rac1 is required for the formation of the three germ layers during gastrulation and knockout mouse is embryonic lethal [40]. In neurons, Rac1 is also crucial for neuronal polarization and axonal growth and differentiation [25,26]. In line with this, the selective forebrain knockout of Rac1 in mice has been shown to cause a global disruption of neuronal growth and development (abnormal lamellipodia formation, deficient migration, impaired axonogenesis, and premature differentiation), which is associated with significant microcephaly [22,26,41]. Rac1 is crucial for the development of the cortical interneurons of GABAergic circuitries and its function is not dispensable in the early stages of this process. Indeed, Rac1 knockout in early pre-migratory neural progenitors in the ventricular zone of the telencephalon is associated with tangential migration failure and morphological defects of developing interneurons [42]. Also, the deregulation of Rac1 activity in neural crest cells has been recently found to negatively affect the differentiation and regionalization of the dopaminergic neuron progenitors in the ventral midbrain [43].

### 2.2. Rac1-Related Disorders

*De novo* missense *RAC1* variants affecting protein function have been associated with a heterogeneous neurodevelopmental disorder (NDD) characterized by variable psychomotor delay and brain malformations, known as Mental Retardation autosomal dominant 48 (MRD48, OMIM # 617751) (Table 1) [26]. The causative variants affect different portions of the protein, although they appear to cluster in proximity to the Switch 1 and 2 domains. The core clinical phenotype of this condition includes mild-to-severe intellectual disability, facial dysmorphism, micro- or macrocephaly, hypotonia, and behavioural disturbances. Cardiac and genitourinary anomalies are less commonly associated features [26]. A wide spectrum of brain MRI abnormalities can be observed in the affected individuals, involving the white matter (corpus callosum hypoplasia or white matter lesions), ventricular and subarachnoid spaces (ventricular enlargement or mega cisterna magna), or cerebral and cerebellar structures. In particular, hypoplasia of the brainstem and hypo-dysplasia of the cerebellar vermis are recurrent, whereas abnormal cortical gyration in the form of polymicrogyria is less frequent [26].

Additional relevant neurodevelopmental conditions are associated with genetic alterations affecting Rac1 function. Among others, a direct link between the underlying pathogenic mechanisms and Rac1 has been suggested in autism spectrum disorders (ASD), schizophrenia, Fragile X syndrome, Rett syndrome, and Huntington’s disease [44]. The Rac1-associated signaling brain network has been involved in the pathogenesis of ASD linked to high-risk genes, such as *AUTS2* (Activator of transcription and developmental regulator, OMIM * 607270), *SHANK3* (SH3 and multiple ankyrin repeat domains 3, OMIM * 606230), and *UBE3A* (Ubiquitin-protein ligase E3A, OMIM * 601623) [45,46,47]. Furthermore, Rac1 appears to be indirectly implicated in the pathogenesis of Rett syndrome, a well-known severe progressive neurodevelopmental condition with significant behavioural abnormalities which is linked to pathogenic variants in *MECP2* (Methyl-cpg-binding protein 2, OMIM * 300005) and *CDKL5* (Cyclin-dependent kinase-like 5, OMIM * 300203). Of note, Rac1 is possibly involved in the pathogenesis of complex neuropsychiatric disorders such as schizophrenia, especially in relation to the specific risk genes *DISC1* (Disrupted in schizophrenia 1, OMIM * 605210), *KALRN* (Kalirin, OMIM * 604605), and *TIAM1* (T-cell lymphoma invasion and metastasis 1, OMIM * 600687) [48,49].

Accumulating scientific evidence has recently emerged in favor of a relevant involvement of Rac1 in cancer progression, especially in brain tumors [50,51]. Most of the supporting studies focus on glioma cells [52,53]. However, the oncogenic role of Rac1 has been highlighted in several distinctive solid tumors, widening the spectrum of Rac-related tumorigenesis. Indeed, genetic alterations involving Rac1 have been identified in a variable proportion of melanomas and lung, uterine, and breast cancers [54]. These findings have paved the way to development of possible targeted therapeutic strategies. For example, the impact of Rac1 function on melanocyte proliferation and motility has suggested innovative treatment strategies based on the modulation of Rac1 localization, GEF-mediated regulation, nucleotide binding, and interaction with downstream effectors [55].

### 2.3. Underlying Pathogenic Mechanisms

The pathophysiological links between *RAC1* variants and the phenotypic manifestations of MRD48 are quite complex and multifaceted. While the (p.Tyr64Asp) variant (NM_018890.4) was found to result in a constitutively activated protein, leading to the formation of lamellipodia in mutant fibroblasts, a dominant negative effect was observed for the (p.Cys18Tyr) and (p.Asn39Ser) variants, associated with abnormalities in fibroblast morphology and zebrafish models [26]. The (p.Val51Met), (p.Pro73Leu), and (p.Cys176Tyr) variants were instead considered to exert context-dependent effects, not displaying clear-cut activating or inactivating effects [26]. According to these findings, the localization of *RAC1* variants may significantly impact the activation state of RAC1, which may range from neutral to dominant negative to constitutively active [26]. *RAC1* variants may therefore affect protein structure and function in a very specific way, making it difficult to draw unique conclusions on their pathogenicity and impact on the phenotypic manifestations observed in human subjects. For example, striking differences in the occipito-frontal circumference measures can be observed among individuals with MRD48, which is unlikely for conditions caused by single nucleotide variants in a specific gene [26]. This peculiarity might reflect the involvement of Rac1 in the mTOR signaling, whose abnormalities are known to be associated with a spectrum of disorders characterized by abnormal growth [26,56]. However, the mechanisms underlying the divergent neurological phenotypes displayed by these same patients can hardly be attributed to similar pathways and still remain elusive. Indeed, while most variants are located in the proximity of Switch 1–2 domains and specific missense variants appear to be associated with distinctive clinical features, no conclusion can be drawn on the existence and the rationale underlying possible genotype-phenotype correlations [26]. This can be in part due to the difficulty of fully characterizing the functional impact of *RAC1* variants, which in some occasions cannot be easily classified as being either constitutively active or dominant negative, and in part to the intervention of additional factors, such as effectors, regulators, or more in general interactors [26,57].

**Figure 4:**
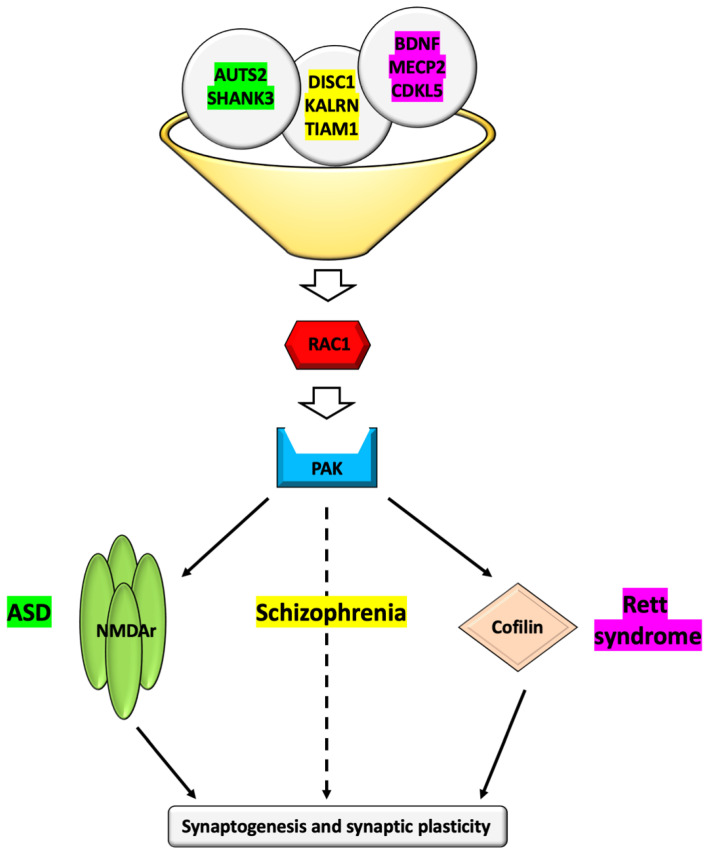
Schematic drawing illustrating the RAC1 signaling pathways involved in the pathophysiology of three main neurodevelopmental conditions: (1) autism spectrum disorder (ASD); (2) schizophrenia; (3) Rett syndrome. The upstream effectors involved in these disorders are indicated in the corresponding color (ASD in green, schizophrenia in yellow, and Rett syndrome in purple). AUTS2 (OMIM * 607270), a transcriptional activator, and SHANK3 (OMIM * 606230), a crucial synaptic scaffolding protein, are involved in ASD. DISC1 (OMIM * 605210), a centrosome-associated scaffold protein regulating several aspects of embryonic and adult neurogenesis, is implicated in the complex pathogenesis of schizophrenia together with the GEFs KALRN (OMIM * 604605) and TIAM1 (OMIM * 600687). In Rett syndrome, the neuronal survival factor BDNF (OMIM * 113505), the chromatin-associated transcriptional regulator MECP2 (OMIM * 300005), and the serine/threonine protein kinase CDKL5 (OMIM * 300203) contribute to the pathogenic model. All the represented proteins converge on RAC1, increasing or decreasing its activity, and modulate the downstream signaling, eventually influencing synaptic formation and plasticity. The dashed line indicates that more complex and multifactorial mechanisms are involved in the pathogenesis of schizophrenia.

Complex and multifaceted mechanisms converging on Rac1 dysfunction are implicated in the pathogenesis of additional conditions with a primary neurological involvement, including ASD, Rett syndrome, and schizophrenia (Figure 4).

In the case of ASD, AUTS2 activates Rac1 through the interaction with several different GEFs, thus promoting the formation of lamellipodia in neuronal cells and, more in general, neuronal migration and neuritogenesis during cortical development [58]. The importance of RAC1 in AUTS2-related corticogenesis is further supported by the rescuing of the abnormal cortical migration phenotype in Auts2-deficient mice by wild-type Rac1 overexpression [58]. As to *SHANK3*, the actin cytoskeleton disorganization in the prefrontal cortex of *Shank3*-deficient mice due to decreased Rac1 and PAK activity has been suggested as the main mechanism underlying the autistic cellular and clinical phenotypes observed in these ASD animal models [59]. In fact, these phenotypic abnormalities and the associated NMDAr dysfunction can be rescued by restoring Rac1/Pak activity [59]. Of note, multiple autism risk genes, such as *UBE3A*, functionally converge on RAC1 and RAC1-dependent memory impairment is implicated in determining the behavioral inflexibility underlying the impaired reversal learning in autistic patients [47].

Although Rett syndrome is a complex medical condition, two high-risk genes, *MECP2* and *CDKL5*, are most commonly involved in affected individuals presenting with the classical or the variant forms of the disease [60]. The pathophysiological link between Rac1 and Rett syndrome is well exemplified by Rac1-mediated dendritic development through brain-derived neurotrophic factor (BDNF) regulation, which represents a common molecular mechanism for MECP2 and CDKL5 pathogenic variants [44]. The BDNF is a protein with a crucial role in synaptogenesis and dendritic spine growth modulation [61,62]. Genetic variants affecting its binding to the tropomyosin-receptor kinase B (TrkB) receptor and the resulting activation of the downstream signaling pathways have been recently associated with neurobehavioural abnormalities [61]. Interestingly, the deregulation of BDNF has been implicated in the decreased spine density observed in the neurons of patients harboring pathogenic *MECP2* variants and RAC1 is thought to act as a downstream signaling effector of BDNF [63,64]. CDKL5 is another crucial regulator of neuronal morphogenesis and it is also required for the BDNF-induced activation of Rac1 [65]. In fact, the disruption of neuronal migration and dendritic arborization resulting from *Cdkl5* knockdown are rescued by wild-type *Rac1* overexpression, further supporting the pathophysiological relevance of this complex interconnection in Rett syndrome [66].

RAC1 can be also involved in the pathogenesis of schizophrenia as the downstream signaling hub molecule for the proteins encoded by risk genes such as *DISC1*, *KALRN*, and *TIAM1* [48,49]. Kalirin-7 is a RAC1 GEF which is downregulated in the prefrontal cortex of schizophrenic individuals [67,68]. Through the interaction with DISC1, this protein controls the intensity and duration of RAC1 activation in response to the N-methyl-D-aspartate receptor (NMDAr) activation [48]. In fact, Rac1 activity in neurons increases as a consequence of DISC1 deficiency and, vice versa, decreases when DISC1 is overexpressed [48]. Another RAC1 GEF, TIAM1, colocalizes with the NR1 subunit of the NMDAr and modulates NMDAr-related RAC1-dependent spine morphogenesis, as highlighted by the reduced spine size observed in association with TIAM1 deficiency [69]. This delicate equilibrium is fundamental to modulate spine growth and size within the brain and, therefore, synaptogenesis and synaptic plasticity [44].

RAC1 is well-known as a central signaling hub required for the transformation mediated by several oncogenes [51,70]. The identification of activating mutations in *RAC1* in samples obtained from melanoma and other malignancies have further supported the crucial driver role of this Rho GTPase in cancer progression [54]. The oncogenic properties of Rac1 are not surprising considering the relevance of this protein in the regulation of essential biological processes, such as proliferation, survival, apoptosis, differentiation, apical-basal polarity, actin cytoskeleton dynamics, inflammatory responses, and production of reactive oxygen species (ROS) [25,55]. Specific studies on brain tumor models have unraveled some underlying mechanisms of RAC1-driven oncogenesis. For example, in a transgenic zebrafish model overexpressing dominant-active (DA) human Akt1 or Rac1G12V, Rac1 was found to play a crucial role in promoting Akt1-driven gliomagenesis [52]. Furthermore, the inhibition of RAC1 resulted in the inhibition of proliferation in cultured human glioblastoma cells, suggesting a significant role for RAC1 in the pathogenesis of human glioblastoma [53]. It is also intriguing that the remarkable impact of Rac1 functions in the regulation of tumoral cell proliferation and migration, angiogenesis, and resistance to chemotherapy is elegantly regulated by its spatio-temporal activation and sub-cellular localization [54,71,72,73,74].

## 3. RAC3

### 3.1. RAC3 Structure, Expression, and Function

The human gene for RAC3 (RAC3, OMIM * 602050) maps to chromosome band 17q25.3 and encodes a 21.4 kDa protein (ENST00000306897.8, ENSP00000304283.4) with a 92% and 89% amino acid identity with RAC1 and RAC2, respectively [18,21]. Most on the divergence between RAC1 and RAC3 resides in the very carboxy-terminal end consisting of the residues 183–192, which is important for subcellular localization and binding to specific regulators [18,21]. RAC3 is specifically expressed in several different regions of the mammalian developing brain, including the human brain. In the latter, it is particularly abundant in the projection neurons of the developing brain, dorsal root ganglia, and spinal cord [18,75]. In comparison to Rac1, exhibiting a ubiquitous expression profile, the expression of Rac3 is strictly and developmentally regulated during mouse brain morphogenesis, despite the only 14 different amino acids between these two Rac proteins [21,75]. Interestingly, the expression profile of Rac3 has been very recently elucidated. Through immunoblotting, it was shown that Rac3 displays a tissue-dependent expression profile in the young adult mouse, being expressed in a developmental stage-dependent manner in the brain [76]. Rac3 was distributed in the cytoplasm but also visualized in axons and dendrites, also partially localizing to the synapses [76]. The immunofluorescence analysis of mouse brain slices showed a strong and moderate distribution of Rac3 in the axons and cytoplasm of cortical neurons at postnatal day P2 and P18, respectively [76]. Furthermore, a comparable expression profile was observed in hippocampal neurons [76]. These findings are consistent with a relevant role of Rac3 during corticogenesis.

Together with Rac1, Rac3 plays a crucial role in the regulation of lamellipodia formation and, therefore, significantly influence the GCs-dependent neuronal migration and development [27,28]. Rac3 triggers the formation of lamellipodia with a comparable efficiency to that of Rac1 (>90%) and its expression in Rac1-deficient cells was found to rescue the lamellipodia-deficient phenotype, thus confirming Rac3 involvement in this relevant aspect of neuronal development [34,77]. Another pivotal role played by Rac3 in neuronal development is the regulation of dendritic spine formation [21]. Although Rac3 knockout hippocampal neurons do not display any obvious alterations, the double Rac1–Rac3 knockout results in a much more severe phenotype, especially characterized by a significant deficiency in the formation of dendritic spines as compared to the single Rac1 knockout [75,78]. In line with this observation, the specificity of Rac3 function of synaptic plasticity is highlighted by the effect of Rac3 re-expression in Rac1–Rac3 double knockout, which results in a significantly more relevant increase in spine size as compared to Rac1 re-expression [79,80]. Eventually, Rac3 was found to specifically interact with β-1 spectrin, a regulator of actin cytoskeleton organization, further substantiating the distinctive role of this protein in comparison to other Rac subfamily members in the modulation of the morphology and the functional dynamic aspects of dendritic spines [81]. Together with the fact that RAC3 appeared relatively late in vertebrate and is absent in lower phyla, this impact on synaptic formation and plasticity led to speculation that this protein might exert a specific role in the regulation of cognitive functions in more advanced life forms, including humans [21,81].

Rac1 and Rac3 play specific roles during brain development that cannot be compensated by the other GTPases. However, to some extent, Rac3 can compensate the lack of Rac1 function in late developmental stages. Together with Rac1, Rac3 is significantly implicated in the development of cortical and hippocampal GABAergic interneurons [78]. Most of these cells originate in the ganglionic eminences (GE) in the developing ventral telencephalon and undergo a complex tangential migration along three main streams (marginal zone, subplate, and subventricular zone), towards their cortical or hippocampal destinations. Here, a switch to radial migration occurs and they populate the target layers [82,83]. While Rac1 primarily impairs the early phase of interneuronal differentiation through a cell- and stage-specific activity in proliferating precursor, the expression of Rac3 is developmentally regulated and displays a peak in correspondence of neurite branching and synaptogenesis [84,85]. Rac1 deletion in the interneuron progenitors from the medial ganglionic eminence (MGE), where most cortical and hippocampal GABAergic interneurons originate, results in relevant migrational defects; these are not observed in postmitotic migratory precursors thanks to a Rac3-mediated compensation [84,86]. This compensation is well-exemplified by the double knockout Rac1/Rac3 mouse model, displaying neurological impairment, epileptic seizures, and a relevant loss of parvalbumin-positive neurons deriving from the MGE in the cerebral cortex and hippocampus [78,87]. In line with the electrophysiological imbalance observed in the cortical and hippocampal circuits of the double knockout mouse model, interneurons lacking both Rac proteins display significant abnormalities of cytoskeletal organization and neurites [87]. The inhibitory function of GABAergic interneurons is indeed crucial in neural circuitry and activity, and their dysfunction leads to an imbalance of excitatory/inhibitory signals resulting in an abnormal brain function [88,89].

### 3.2. Rac3-Related Disorders

A novel neurodevelopmental condition characterized by structural brain anomalies and dysmorphic facies (NEDBAF, OMIM #618577) has been recently associated with pathogenic variants in *RAC3* [90,91]. Global developmental delay (GDD) leading to severe to profound intellectual disability (ID), abnormal muscle tone, variable neurological involvement, and structural brain abnormalities are the cardinal features of this emerging disorder [90,91]. Unlike Rac1-related MRD48, microcephaly is not common and macrocephaly has never been observed in affected individuals [26,90,91,92]. With the refinement of the phenotypic spectrum, additional clinical manifestations have been included in the core phenotype. In particular, affected individuals present with recurrent facial dysmorphic features, feeding difficulties leading to failure to thrive, epilepsy, and respiratory problems [92]. Neurological manifestations include dyspraxia, spasticity, behavioural disturbances and stereotyped movements [92]. Not infrequently, syndromic features can be observed, such as genitourinary and endocrinological abnormalities [92]. Of note, while Rac3-specific behavioural abnormalities (i.e., hyperactive behaviour) not linked to cognitive deficiency were reported in the knockout mouse model, these features are quite rare in human *RAC3* patients [92].

In line with the extremely relevant role played by RAC3 in neuronal development, brain MRI findings are particularly relevant in these patients. White matter abnormalities are among the most common alterations in the form of corpus callosum agenesis/hypoplasia and thinning of subcortical white matter [90,91,92]. Malformations of the cortical development are a cardinal feature present in a large proportion of reported individuals, especially consisting of polymicrogyria and dysgyria [90,91,92]. Brain MRI may also show gray matter heterotopias, cerebellar dysplasia, and variable abnormalities of the brainstem [90,91,92].

### 3.3. Underlying Pathogenic Mechanisms

Rac3 participate in various aspects of neuronal development, especially axon and dendrite formation, neuritogenesis, regulation of migration, and synaptogenesis [78,79,84,87,93]. The reported *RAC3* variants appear to negatively affect the protein basic functions, leading to an impairment of actin cytoskeletal organization and signal transduction [90,91]. However, the exact molecular mechanisms implicated in the pathogenesis of a complex condition such as NEDBAF have been long remained elusive.

Through in silico protein modeling, *RAC3* variants have been suggested to variably alter the conformation and function of the main functional domains of RAC3, Switch I and Switch II [90]. In particular, the p.(Pro29Leu) variant (NM_005052.3) in the Switch I domain was predicted to cause a conformational change due to the introduction of a hydrophobic sidechain and the increased loop flexibility, likely leading to an impaired flexibility of the domain in a comparable way to the p.(Pro29Ser) variant in *RAC1* [90,94,95,96]. As to the *RAC3* variations affecting the Switch II domain, the (p.Gln61Leu) was predicted to disrupt the H-bond that RAC3 forms with the phosphate group of phosphoaminophosphonic acid-guanylate ester (GNP) and destabilize the small 3(10)-helix at residues 62–64 [90]. An alteration of the conformation of this 3(10)-helix was also expected for the (p.Glu62Lys) variant due to electric changes in the charge of the sidechain [90].

Very recently, the pathophysiological aspects of causative *RAC3* variants in NEDBAF patients have been elucidated [92]. In particular, it was observed that the 11 tested *RAC3* variants acted as GTP-bound variably active forms of the protein, in contrast with what has been previously reported on *RAC1* variants [26,92]. These findings led to the assumption that a variant-dependent spatio-temporal dysregulation of common downstream effectors, in terms of hyperactivation or inhibition, may be responsible of the divergent phenotypes associated with *RAC1* and *RAC3* variants [26,92]. Since Rac1 and Rac3 mainly diverge in the C-terminal polybasic region and an adjacent CAAX box, which are essential for the proper localization of the proteins within the cell, their diverse subcellular localization has been suggested as a possibly relevant cofactor in determining the differences between *RAC1*- and *RAC3*-related pathogenic mechanisms and clinical phenotypes [92]. RAC1 and RAC3 cooperate in neuronal signaling networks, which are crucial for synaptic function, which is in favor of their crucial role in the pathophysiology of the neurodevelopmental impairment in human patients [21,97]. However, the observation that brain size abnormalities are predominant in patients harboring *RAC1* variants whereas *RAC3* subjects more commonly display malformations of cortical development (polymicrogyria, heterotopia and dysgyria) suggest that RAC1 likely plays a major role in neurogenesis and/or apoptosis, whereas RAC3 is primarily involved in neuronal migration [26,44,92].

The Switch II domain is essential for the physical interaction of Rac proteins with the GEFs and GAPs, and represents a mutational hotspot for both RAC1- and RAC3-related disorders [26,92]. In this regard, the pathophysiology of *RAC3* variants localized to this relevant functional domain most likely involves an aberrant interaction with regulatory proteins in a variant-specific and context-dependent manner [92]. Although *RAC3* variants have been shown to favor an activated conformation of the protein, suggesting an underlying gain-of-function mechanism, further studies on the interaction of RAC3 mutants with downstream effectors revealed unexpected results. In particular, the activation state of mutants did not directly correlate with their affinity towards the RAC-binding regions (RBRs) of different downstream effectors, including PAK1, MLK2, IRSp53, N-WASP, ROCK, and RTKN [92]. In line with this observation, it is possible that the abnormal upregulation of RAC3 effectors occurs in a variant-type- and context-dependent manner, so that a definite variant may act as an activator for some downstream signaling pathways while exerting negative dominant effects for others [92]. Eventually, this intricate pathophysiological background may pave the way to speculation on possible genotype-phenotype correlations, based on the supposition that the specific quantitative and qualitative effect of single variants on intracellular signaling networks may correlate with the divergent phenotypic manifestations observed in different affected individuals [92].

## 4. Implications of Rac Proteins Effectors and Regulators in NDDs

**Figure 5:**
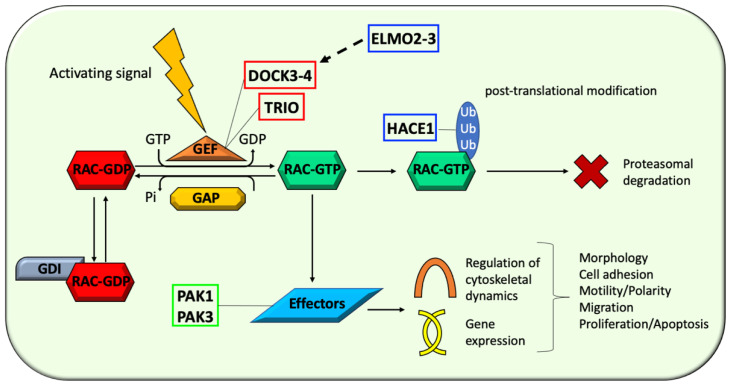
Schematic drawing illustrating selected GEFs (red), regulators (blue), and effectors (green) of RAC proteins which have been associated with human conditions with a primary neurological involvement. The represented categories include: (1) GEFs: DOCK3, DOCK4, and TRIO; (2) regulators: ELMO2, ELMO3, and HACE1; (3) effectors: PAK1 and PAK3. GEFs plays a crucial role in the ‘off-on’ switching of Rac GTPases, regulators are involved in the fine modulation of aspects of Rac proteins function (ELMO2 and ELMO3) and metabolism (HACE1), and effectors are primarily responsible of the downstream effects of Rac signaling (PAK1 and PAK3).

A growing interest has recently emerged on the involvement of effectors and regulators of RAC1 and RAC3 function in the pathogenesis of human disorders. Among them, a particularly relevant pathophysiological role in neurological conditions is played by GEFs (TRIO, DOCK3, and DOCK4), regulators (HACE1 and ELMO3), and effectors (PAK1 and PAK3) (Figure 5). The involvement of Rac proteins in the pathogenesis of these conditions is based on their functions towards RAC signaling. However, scientific evidence in support of a direct involvement of RAC dysfunction still lacks in most cases and will be highlighted in the manuscript when available. A further relevant aspect to consider when discussing these pathophysiological mechanisms is that RAC effectors and GEFs are highly dynamic multi-functional molecules which regulate neuronal function through diverse and complex pathways, often making it difficult to predict the extrapolated linear cause-effect relationships.

### 4.1. Activators: Unbalanced RAC Proteins Activation by Guanine Nucleotide-Exchange Factors (GEFs), Such as TRIO, DOCK3, and DOCK4, Is Implicated in NDDs Pathophysiology

The activation of Rho proteins is mediated by specific GEFs catalyzing the exchange of GDP for GTP, allowing the fine regulation of their biological activity through multiple different pathways. Depending on the localization of the catalytic activity, two subfamilies of Rho GEFs have been recognized: GEFs with the Dbl homology (DH) domain, containing a conserved domain between yeast Cdc24 and Dbl; GEFs with the CDM and Zizimin homology (CZH) domain or Dock180-related proteins, containing two highly homologous regions mediating nucleotide exchange on Rho GTPases known as Dock homology region (DHR) 1 and 2 [98,99,100,101]. Among the more than 70 Rho-interacting GEFs encoded in the human genome, TRIO, DOCK3, and DOCK4 are crucial regulators of Rac proteins activity and plays a significant pathophysiological role in Rac-related NDDs.

#### 4.1.1. Triple Functional Domain Protein (TRIO)

The triple functional domain protein (TRIO, OMIM *601893) is a highly conserved GEF containing three functional domains: two GEF domains and a serine/threonine kinase domain. The GEF domain 1 (GEFD1) is crucial for RAC1 and RHOG regulation, whereas the second domain (GEFD2) is involved in the regulation of RHOA [102,103]. Additionally, this protein contains several accessory motifs, among which N-terminal spectrum repeats [103]. TRIO is highly expressed in the developing brain, where it exerts a pivotal role through the activation of RAC1. In fact, TRIO is involved in several fundamental processes as a primary regulator of cytokinesis, including neuronal migration, azonal guidance and growth, and dendritogenesis, thus being directly involved in synaptic formation and function [104,105,106]. Trio knockout is lethal in mouse embryos and is associated with skeletal muscle abnormalities and abnormal development of the nervous system, whereas induced haploinsufficiency in early embryogenesis causes learning and social deficits combined with impaired motor coordination [107,108].

In humans, pathogenic variants in TRIO cause variable neurodevelopmental phenotypes with ASD features which can be mainly classified into two distinct forms of intellectual disability with micro- or macrocephaly: autosomal dominant intellectual developmental disorder 44, with microcephaly (MRD44, OMIM #617061), and autosomal dominant intellectual developmental disorder 63, with macrocephaly (MRD63, OMIM #618825). MRD 44 is characterized by psychomotor delay, dysmorphic features, minor skeletal abnormalities, prominent behavioural disturbances, and recurrent infections [109,110,111,112,113]. Patients with MRD63 similarly present with developmental delay, facial dysmorphism, behavioral abnormalities (including stereotypies), and skeletal features, but also display a peculiar association of poor growth and macrocephaly (Table 2) [111,112].

The pathophysiological mechanisms involved in the origin of these two distinct neurodevelopmental conditions are based on the type, localization, and functional impact of TRIO variants. While the nonsense variants leading to truncated transcripts or haploinsufficiency are scattered along the DNA sequence, missense variants cluster in two specific mutational hotspots, the GEFD1 and the seventh spectrin domain [107,109,111,112,114]. Most GEFD1 mutants lead to a decreased TRIO-mediated RAC1 activation, whereas others have been found to affect glutamatergic transmission [107,112,114]. Conversely, missense variants in the seventh spectrin domain are associated with an hyperactivation of RAC1 through an indirect mechanism [111]. Indeed, while GEFD1 is directly implicated in RAC1 activation, pathogenic variants in the spectrin mutational hotspot likely impair TRIO folding and signaling due to a perturbation of mutual arrangement of the α helices and, possibly, GEFD1-RAC1 intramolecular binding dynamics [41,111]. Intriguingly, these two mutational hotspots are associated with distinctive neurodevelopmental phenotypes from the clinical perspective. While loss-of-function variants in GEFD1 cause a milder intellectual disability with microcephaly (MRD44), gain-of-function variants in the seventh spectrin domain cause a more severe cognitive impairment with macrocephaly (MRD63) [111].

#### 4.1.2. Dedicator of Cytokinesis 3 and 4 (DOCK3 and DOCK4)

Dedicator of cytokinesis 3 and 4 (DOCK3, OMIM * 603123; DOCK4, OMIM * 607679) are members of the DOCK-B subgroup of the dedicator of cytokinesis (DOCK) family, evolutionary conserved GEFs for the Rho GTPases RAC and CDC42 [115,116]. DOCK proteins are characterized by a DOCK homology region (DHR) 1 domain, responsible for phospholipids binding, and DHR2 domain, which exerts the GEF activity [115,116]. Cell adhesion and migration, and the regulation of actin cytoskeleton are among the crucial biological processes regulated by these GEFs, which are also implicated in cancer invasion [115]. Both DOCK3 and DOCK4 are highly expressed in the brain and are directly implicated in several processes underlying the development and function of neuronal cells, microglia and Schwann cells [117,118].

Emerging scientific evidence has highlighted the pathophysiological links between the dysfunction of DOCK proteins and both neuropsychiatric and neurodegenerative conditions such as ASD, schizophrenia, and Parkinson’s and Alzheimer’s diseases [118]. More specifically, biallelic pathogenic variants in *DOCK3* leading to a loss of function have been recently involved in the pathogenesis of a neurodevelopmental disorder with impaired intellectual development, hypotonia, and ataxia (NEDIDHA, OMIM # 618292) [116,119]. This condition is characterized by cognitive impairment associated with dysmorphic features, hypotonia, and minor skeletal abnormalities, including mild short stature [116,119]. The disruption of DOCK4 function caused by two distinct variations (Exon 27–52 deletion and the missense variant (p.Arg853His)) leading to a decreased ability to activate Rac1 has been instead implicated in the pathogenesis of dyslexia and ASD with poor reading abilities [120,121,122].

### 4.2. Regulators: Dysfunctional RAC Acivity Modulation by Regulatory Proteins, Such as HACE1, ELMO2, and ELMO3, Contributes to NDDs Pathogenesis

Although the activation operated by GEFs is critical for Rac proteins function, their biological activity is also less directly affected by the intervention of further specific proteins, whose role is to regulate distinct and relevant steps of Rac metabolism and function. Among these, ubiquitination (HACE1) and scaffolding-mediated modulation of GEFs activity (ELMO2 and ELMO3) are of particular interest in relation to their involvement in the pathogenesis of neurodevelopmental conditions.

#### 4.2.1. HECT Domain- and Ankyrin Repeat-Containing E3 Ubiquitin Ligase 1 (HACE1)

HACE1 (OMIM * 610876) encodes a HECT domain- and ankyrin repeat-containing E3 ubiquitin ligase that regulates the activity of cellular GTPases, including Rac1 [123]. This ubiquitin ligase recruits the E2 enzyme UBCH7 to the ubiquitinate HACE-1 specific target proteins, favoring the subsequent degradation by the 26S proteasome [124]. HACE1 has been primarily implicated in tumorigenesis and cancer progression as a tumor suppressor gene, according to its inactivation in Wilms’ tumors and other cancers as well as its reduced expression in colon and gastric cancers [124,125,126]. However, it is highly expressed in the brain and targets the active form of Rac1, suggesting a possible relevant role in brain development and function [127]. HACE1 also interacts with other members of the Rab family [128,129]. Indeed, it is recruited to the Golgi by Rab1 for the disassembly of the mitotic complex and promotes the Rab11a-dependent recycling of β2-adrenergic receptor (β2-AR) [128,129].

Biallelic loss of function variants in *HACE1* have been associated with a complex neurodevelopmental condition known as spastic paraplegia and psychomotor retardation with or without seizures (SPPRS, OMIM # 616756) [123,130,131,132]. This disorder is characterized by global psychomotor delay, hypotonia, spastic ataxia, dystonia, seizures, and syndromic features (short stature, overweight, hearing loss, microcephaly, retinal dystrophy, abnormal genitalia, kyphosis, and other skeletal deformities) [123,130,131,132]. Neuroimaging findings include corpus callosum hypoplasia, delayed myelination, decreased white matter volume, and cerebral atrophy [123,130,132]. Of note, functional studies investigating the underlying pathophysiological links showed that *Hace1* knockout mice models phenocopy the key clinical features observed in human patients, both in terms of brain developmental abnormalities and neurological features [132].

In line with HACE1’s role in Rac1 ubiquitination, increased levels of the active form of Rac1 were observed in knockout models and patient-derived fibroblasts with loss of *HACE1* expression, which indeed showed a much faster migration in comparison to wildtype [28,40,132]. Interestingly, either overexpression or downregulation of RAC1 causes a reduction of dendritic spines, which is recapitulated by *Hace1* knockout mouse [132]. Furthermore, RAC1 can regulate reactive oxygen species (ROS) levels, and SPPRS patient–derived fibroblasts exhibited an ROS upregulation [40,132]. Taken together, these findings support the increased Rac1 activity due to deficient degradation as the main pathophysiological driver in SPPRS [132].

#### 4.2.2. ELMO/CED12 Domain-Containing Protein 2 and 3 (ELMO2 and ELMO3)

DOCK-family proteins are RAC-specific GEFs playing pivotal roles in axono- and dendritogenesis, neuronal differentiation, and cell polarity [115,116]. DOCK1–5 members interact and can form a complex with the members of the Engulfment and Cell Motility (ELMO) family of scaffold proteins, including ELMO1, ELMO2 and ELMO3 [133,134]. The ELMO/DOCK complex exists in two activity states: a resting state, in which the complex has a closed confirmation with an auto-inhibition mediated by specific intramolecular contacts; and an active state, in which the complex in an open conformation promotes the GTP-loading od Rac1 [135]. The ELMO/DOCK pathway has been implicated in several aspects of central nervous system development [117,136,137]. In particular, the complexes between DOCK proteins and ELMO1 and ELMO2 have been implicated in neuritogenesis and dendrite formation, whereas the link between ELMO3 and neuronal development have long been more elusive [117,136,137].

Very recently, compound heterozygous variants in *ELMO3* (OMIM * 606422) have been associated with psychomotor delay and ASD in a five-year-old male child [138]. Both the reported variants (p.Ser385Cys and p.Val337Ile) (NM_024712.5) were found to markedly impair the ability of the ELMO3/DOCK1 complex to promote RAC1-GTP-loading. Furthermore, impaired migration and invasion was observed in ELMO3 mutant cells, suggesting that the lower activity of RAC1 might be a relevant contributor to the neuronal dysfunction underlying the ELMO3-related neurodevelopmental phenotype [138]. Loss of function variations in *ELMO2* (OMIM * 606421), in the form of small insertions or deletions have been associated with a rare malformative condition known as vascular malformation, primary intraosseous (VMPI, OMIM # 606893) [139]. However, a homozygous missense variant in *ELMO2* has been recently reported in a young girl with intellectual disability, seizures, dysmorphic features, and associated syndromic manifestations suggestive of Ramon syndrome [140]. The (p.Ile606Ser) variant (NM_182764.2) localizes to the atypical Pleckstrin homology domain of ELMO2 involved in the interaction with the DOCK proteins, suggesting that a dysregulation of the activity of these GEFs possibly leading to an abnormal Rac1 function might be a relevant underlying pathogenic mechanism in *ELMO2*-related disorder [140,141]. These emerging disease associations support a role of *ELMO* genes in the pathogenesis of human conditions with a primary neurological involvement.

### 4.3. Effectors: The Emerging Role of RAC Proteins Effectors, Such as PAK1 and PAK3, in Human NDDs

Once in the active state, Rac GTPases can activate several distinct downstream effectors, which ranges from kinases to proteins associated with actin cytoskeleton [15]. These effectors act within diverse biological pathways which can be classified in two main categories: regulation of cytoskeletal dynamics and gene expression modulation (Figure 5) [15]. In the vast universe of Rac effectors, a growing interest has developed with regard to two kinases with an emerging pathophysiological link with human neurodevelopmental phenotypes, PAK1 and PAK3.

#### p21 Protein-Activated Kinase 1 and 3 (PAK1 and PAK3)

The p21 protein-activated kinase 1 and 3 (PAK1, OMIM *602590; PAK3, OMIM *300142) are members of the group I of the family of PAK proteins, serine/threonine kinases acting as effectors of CDC42 and RAC proteins, and playing a pivotal role in signal transduction and cellular regulation [142,143]. The kinase activity of PAK1 and PAK3 is autoinhibited by a homodimeric conformation in which the N-terminal portion of one monomer binds the catalytic domain of the other monomer leading to its inhibition [144]. The binding of activated RAC proteins or CDC42 to the GTPase binding domain of PAK1 and PAK3 dissociates this dimerization and allows the autophosphorylation, paving the way to the activation loop of PAK catalytic activity [144]. PAK proteins act as effectors of CDC42 and RACs, thus being directly involved in mediating their crucial regulatory effects on the cytoskeleton, such as the regulation of cell shape and motility, and the formation of filo- and lamellipodia [145,146]. PAKs are highly expressed in the nervous system, and both PAK1 and PAK3 have been directly implicated in the regulation of brain size and function in mice, with a relevant role in synapse function and plasticity [147,148].

Pathogenic variants in *PAK1* cause an autosomal dominant form of intellectual developmental disorder with macrocephaly, seizures, and speech delay (IDDMSSD, OMIM # 618158). This condition is also characterized by facial dysmorphic features and abnormal behaviour [149]. *PAK3* variants are instead associated with X-linked intellectual developmental disorder 30 (XLID30, OMIM # 300558), a disorder characterized by mild to severe cognitive impairment, dysmorphic features, and epilepsy [150,151,152,153]. Furthermore, affected individuals present with severe neuropsychiatric manifestations, including anxiety, hyperactivity, aggressivity, and psychosis [150,151,152,153]. Brain MRI abnormalities have also been reported in two affected fetuses, including agenesis of the corpus callosum agenesis, abnormal conformation of the brainstem and pyramidal tract, hydrocephalus, and subependymal cysts [154,155]. Symptomatic females are rare and present with intellectual disability and behavioural disturbances, occasionally associated with brain abnormalities and abnormal eye movements [156].

PAK1 And PAK3 mediate relevant effects of RAC signaling, including the regulation of cell shape, motility, and adhesion, as well as the formation of lamellipodia and filopodia [145,146]. As such, the dysregulation of their activity can lead to an abnormal efficiency of RAC signaling, leading to abnormalities in several cell processes [3,10]. However, the underlying mechanisms might be complex than expected based on a simple loss- or gain-of-function model and mostly remain elusive. In particular, IDDMSSD is caused by dominantly-acting, gain-of-function pathogenic variants in *PAK1* which likely mimic a hyperactive downstream RAC signaling, leading to the consequences observed in case of RAC overexpression [20,21]. Conversely, *PAK3* variants causing XLID30 are predicted to act through more complex and yet to be fully elucidated mechanisms [149,150,151,152,153]. This is exemplified by the (p.Arg67Cys) variant in *PAK3* (NM_001128168.3), which decreases PAK3 binding to CDC42 but increases the binding to Rac1 [157]. In this regard, the abnormal function of Rac1 might underlie the deficient dendritic outgrowth and spine maturation observed during hippocampal neurogenesis in the mutant *Pak3*-R67C mouse [20,21,157].

## 5. Conclusions

The members of the RAC subfamily of RHO GTPases play pivotal roles in the crucial processes of several different cell types. The abnormal modulation or disruption of their function is implicated in the pathogenesis of distinctive human conditions with abnormal brain development and neurological impairment, collectively definable as neuro-RACopathies. The underlying pathophysiological mechanisms are complex and often involve the hyperactivation of RAC proteins or, conversely, dominant negative effects resulting from genetic variations in the *RAC1* and *RAC3* genes. However, variants that are not clearly predicted to directly affect protein function in one of these ways may still affect protein function through alternative mechanisms, such as affecting the interaction of RAC proteins with their regulators or downstream effectors. To further complicate the picture, though making it still more intriguing, variants in the genes encoding these RAC-related interactors significantly contribute to the pathogenesis of additional neurological conditions themselves. In this puzzling panorama, further details on the molecular machinery driving the pathogenesis of neuro-RACopathies still remain to be elucidated. The better understanding of how RAC proteins dysfunction determines the different clinical conditions might prompt the way to the development of more appropriate management strategies and, possibly, innovative therapeutic strategies as well.

## Figures and Tables

**Table 1:** Synopsis of Rac proteins and related disorders.

Rac Protein	Tissue Distribution	Human Gene (OMIM)	Associated Medical Conditions (OMIM)
RAS-related c3 botulinum toxin substrate 1; RAC1	Ubiquitous	*RAC1 *(* 602048)	Mental retardation, autosomal dominant 48; MRD48 (# 617751)
RAS-related c3 botulinum toxin substrate 2; RAC2	Hematopoietic system	*RAC2 *(* 602049)	Immunodeficiency 73a with defective neutrophil chemotaxis and leukocytosis; IMD73A (# 608203);
			Immunodeficiency 73b with defective neutrophil chemotaxis and lymphopenia; IMD73B (# 618986);
			Immunodeficiency 73c with defective neutrophil chemotaxis and hypogammaglobulinemia; IMD73C (# 618987)
RAS-related c3 botulinum toxin substrate 3; RAC3	Nervous system	*RAC3 *(* 602050)	Neurodevelopmental disorder with structural brain anomalies and dysmorphic facies; NEDBAF (# 618577)

**Table 2:** Synopsis of neuro-RACopathies associated with RAC proteins interactors.

Rac Proteins Interactor.	Human Gene (OMIM)	Category	Pathophysological Mechanism in Relation to Rac Proteins	Associated Medical Conditions (OMIM)
Triple functional domain protein	*TRIO* (* 601893)	GEF	LoF mechanism: ↓ TRIO-mediated RAC1 activation (MRD44); GoF mechanism: indirect ↑ activation of RAC1 (MRD63)	Intellectual developmental disorder, autosomal dominant 44, with microcephaly (MRD44, # 617061); Intellectual developmental disorder, autosomal dominant 63, with macrocephaly (MRD63, # 618825)
Dedicator of cytokinesis 3	*DOCK3* (* 603123)	GEF	Lof mechanism: ↓ GEF-mediated RAC activation	Neurodevelopmental disorder with Impaired intellectual development, hypotonia, and ataxia (NEDIDHA, # 618292)
Dedicator of cytokinesis 4	*DOCK4* (* 607679)	GEF	Lof mechanism: ↓ GEF-mediated RAC activation	Dyslexia and ASD with poor reading abilities
Hect domain- and ankyrin repeat-containing e3 ubiquitin protein ligase 1	*HACE1* (* 610876)	Regulator	Lof mechanism: ↓ ubiquitination leading to ↑ Rac1 activity	Spastic paraplegia and psychomotor retardation with or without seizures (SPPRS, # 616756)
Engulfment and cell motility gene 2	*ELMO2* (* 606421)	Regulator	Possible LoF mechanism: abnormal interaction with DOCK proteins	Ramon syndrome (# 266270)
Engulfment and cell motility gene 3	*ELMO3* (* 606422)	Regulator	Lof mechanism: ↓ RAC1-GTP-loading by ELMO3/DOCK1 complex	Psychomotor delay and ASD
p21 protein-activated kinase 1	*PAK1* (* 602590)	Effector	GoF mechanism: ↑ activation of Rac signaling pathway	Intellectual developmental disorder with macrocephaly, seizures, and speech delay (IDDMSSD, # 618158)
p21 protein-activated kinase 3	*PAK3* (* 300142)	Effector	GoF mechanism: ↑ Rac1 binding and activation	Intellectual developmental disorder, x-linked 30 (XLID30, # 300558)

Abbreviations: autism spectrum disorder (ASD); guanine nucleotide exchange factors (GEF); gain-of-function (GoF); loss-of-function (LoF); Online Mendelian Inheritance in Man (OMIM).

## Data Availability

No new data were created or analyzed in this study. Data sharing is not applicable to this article.
